# A Thermodynamic Theory of Axon Guidance: Navigation Through High-Entropy Signaling States

**DOI:** 10.64898/2026.09.22.752727

**Published:** 2026-10-14

**Authors:** William G. Wadsworth

## Abstract

Precise neural circuit formation requires growth cones to integrate multiple, sometimes competing, guidance signals into persistent yet adaptable movement. Here, we propose a theoretical framework that recasts axon guidance as a thermodynamically regulated computation. An artificial neural network, trained on in vivo genetic data and associated outgrowth patterns, maps the microstates of a localized guidance signaling network to macroscopic outgrowth behaviors. Using the trained model, we simulated axon pathfinding through extracellular gradients of molecular cues and created dynamic entropic landscapes. These results reveal that growth cones navigate high-entropy ridges that preserve plasticity. Navigation along these ridges supports persistent extension, whereas turning and branching occur near boundaries between competing entropic macrostates, consistent with transitions in the cytoskeletal machinery that controls growth-cone movement. More broadly, the framework proposes that robust biological patterning can emerge from microscopic variability when signaling networks operate near boundaries between competing behavioral states.

**Significance Statement:**

How do complex biological systems remain robust yet adaptable? We address this fundamental question by conceptualizing axon guidance as a thermodynamic process. By embedding a neural-network predictive model of signal integration within a statistical physics framework, we show computationally that migrating axonal growth cones need not passively settle into stable states, but can instead navigate fluctuating, high-entropy states. This thermodynamic strategy reconciles persistence with plasticity, allowing directed movement while keeping growth cones poised near critical boundaries where small biochemical fluctuations can trigger rapid turning or branching. More broadly, this framework suggests a general principle by which complex cellular systems use network degeneracy and fluctuations to coordinate robust behavior with rapid adaptation during migration, pattern formation, and tissue development.

